# Reconstructing Multilingual Development Research: Shifting from a Monolingual Bias and Toward a Developmental Systems Framework

**DOI:** 10.3390/bs16030473

**Published:** 2026-03-22

**Authors:** Marissa A. Castellana, Viridiana L. Benitez

**Affiliations:** Department of Psychology, Arizona State University, Tempe, AZ 85287-1104, USA

**Keywords:** monolingual bias, multilingualism, developmental systems framework, interaction, context

## Abstract

Multilingual research offers a unique window into the diverse developmental trajectories of language and cognition; yet this research has largely been built on a monolingual framework. Here, we first describe how a monolingual bias has limited theory construction and research on the multilingual experience. We then apply a developmental systems framework to understand the multilingual experience, shifting the field away from a monolingual bias toward centering the lived language experiences of multilingual children. At the center of our framework are the moment-to-moment, multimodal, and dynamic interactions between children, their social partners, and environment. Contributing to interaction dynamics are child and social partner characteristics (cognition, motivation, and experiences), as well as contextual factors (activities, places, and policies) that can shape multilingual exposure. Cultural practices, values, and beliefs, as well as developmental time at the micro level (seconds, hours, days) and the macro level (weeks, months, and years), permeate all levels of the framework. Our proposal reveals important avenues of future research, including (1) understanding the dynamic coordination of multimodal behaviors and languages within interactions, (2) how experiences specific to minoritized communities (e.g., language discrimination) shape interaction dynamics, (3) how the temporal patterns of language experience at the micro level contribute to long-term multilingual exposure, and (4) understanding experiences of different multilingual communities within and across communities. Use of this framework can advance knowledge of the contexts enriching multilingual experiences and reconstruct multilingual development research for the benefit of multilingual learners.

## 1. Introduction

Multilingualism, the knowledge of two or more languages, is a common human experience. The prevalence of multilingualism across the globe has been increasing tremendously over the past few decades (Escobar, 2013; Eurostat, 2021; Liddicoat & Kirkpatrick, 2020; Lo Bianco, 2015; Migration Policy Institute, 2023; Posel & Zeller, 2016). Currently, more than half of the global population is estimated to be multilingual (Grosjean, 2021), a large proportion of which comes from minoritized communities (e.g., immigrants, ethnic and/or racial minorities, native/indigenous groups; Language Resource Center, 2022; Migration Policy Institute, 2023). Research on multilingualism and with multilingual populations can offer a unique window into the diverse developmental pathways of language and cognition. Yet, multilingual research remains underrepresented within the field of language acquisition (Kidd & Garcia, 2022). Further, the most foundational questions on multilingual development, which involve measuring and defining multilingualism, are still being debated today, especially in early childhood when language knowledge is emerging. Without precise answers to these questions, it is difficult to identify how to best promote multilingual learners’ path to success in language and cognition.

In this paper, we argue that in order to make progress in multilingual development research, we must reframe one of the most basic questions in the field: what is the multilingual experience? We define multilingual experiences as those that expose a learner to two or more languages, and we define monolingual experiences as those that expose learners to a single language. We use exposure to refer to any experience with languages, including the interactional use of or communicative engagement with languages (e.g., hearing or responding to child-directed speech) and meaningful ambient presence of languages (e.g., overheard speech directed to others around the child or from media), as these forms of exposure can shape opportunities for language learning. We begin by showing how the monolingual bias has created a significant barrier to the study of the multilingual experience. We then present a developmental systems approach for centering and understanding multilingual language experiences to move the field beyond the monolingual bias. We end with key areas of future research highlighted by the framework and recommend practices that serve to reconstruct multilingual research to better understand the multilingual experience.

### 1.1. The Monolingual Bias Constrains Multilingual Research

There is a pervasive monolingual bias in the research that is promoted, funded, and dominates the field of psychology (e.g., Sánchez-Pérez & Manzano-Agugliaro, 2021). The monolingual bias is the ideology that monolingualism is the norm, and multilingualism is the exception, privileging the status of monolingualism (Barratt, 2018; Flores, 2016; López et al., 2021; Manalili, 2021). Critically, a major consequence of the monolingual-as-default assumption is that a monolingual framework is typically applied to research framing multilingual populations, yielding unjust comparisons that favor monolinguals or use them as a control group (Leivada et al., 2023; Rothman et al., 2023). The consequences of this assumption are important: the unique experiences that shape multilinguals’ language development are often not reflected in the construction of pedagogy, policy, and regulations, which place multilinguals at a disadvantage (Barratt, 2018; Manalili, 2021; Vaid & Meuter, 2016).

While a monolingual bias exists in the fields of linguistics, education, and psychology (e.g., Barratt, 2018; Flores, 2016; López et al., 2021; Manalili, 2021; Vaid & Meuter, 2016), here, we focus on the field of language development specifically (Genesee, 2022). The field of language development should not only recognize that the monolingual bias exists so that we can reject hegemonic monolingualism (López et al., 2021), but we also need to understand *where* research perpetuates this bias in order to dismantle it (Syed et al., 2018). We argue that the monolingual bias has produced an overrepresentation of research emphasizing the percentage of language exposure as the defining feature of multilingual environments, which distorts our understanding of the rich and diverse multilingual experiences that could build multilingual knowledge.

### 1.2. Overemphasizing Percentage of Language Exposure

Children learn the language(s) of their community (Heath, 1983; Hoff, 2006). As such, a large literature has focused on understanding the early experiences that monolingual children have with language, with an emphasis on the quantity of language exposure as a critical component in a child’s environment that supports their language acquisition (e.g., Anderson et al., 2021; Hoff & Naigles, 2002; Hurtado et al., 2008; Huttenlocher et al., 1991; Weisleder & Fernald, 2013). Because the field views monolingualism as the default experience, the focus on quantity has translated to multilingual research, resulting in percent exposure being the central metric used to describe multilingual experience and its effects on development.

In particular, the *percent exposure* metric has been frequently used to not only characterize the multilingual experience, but also to define who is and is not multilingual. In fact, the percent exposure metric is the most reported feature to describe early multilingual experience (Surrain & Luk, 2019), despite it being *just one* of many metrics of the language environment. We believe that the overemphasis on percent exposure stems from the monolingual bias. Specifically, the monolingual child, exposed to a single language, spends 100% of their time listening to that language, whereas multilingual children share their time between their languages. Viewed through the lens of monolingualism, multilingual children will always hear any one of their languages a smaller percentage of time than a monolingual hears their single language. Through that lens, using percent exposure as a metric of multilingual experience may reflect an attempt to reassemble the multilingual child into one whole equivalent of a monolingual child.

This fixation on percent exposure has obscured our exploration of other aspects of language environments (e.g., activity, timing, social partners) and has detrimentally narrowed our view of the multilingual experience. Here, we demonstrate that the use of this percent exposure metric fails to push the field forward with regards to understanding the multilingual experience in three key ways: (1) there is no consensus for using this metric to classify learning environments, (2) it has led to the systematic exclusion of some multilingual experiences from research, and (3) it does not fully capture meaningful differences in quantitative or other features across environments.

**(1) Variability in defining monolinguals vs. multilinguals.** First, there is no consensus with regard to how much exposure to a given language is enough for a child to be considered growing up in a monolingual or multilingual environment. The majority of studies do not document their participants’ language status (often defined as monolingual or bilingual; Surrain & Luk, 2019), and the studies that do report participant language status implement varying definitions of monolingual vs. multilingual categories.

Through a scoping review, Rocha-Hidalgo and Barr (2023) examined the variability in percent exposure metrics to classify infants under 36 months as exposed to a monolingual vs. bilingual environment across 127 articles (167 studies). Monolingual exposure was most often defined as experiencing a minimum of 90% in one language, although studies varied widely, ranging from 65% to 100% exposure (meaning that children could be classified as monolingual if they received 0% to 35% exposure to an additional language). On the other hand, bilingualism was most frequently defined as receiving 20% or 25% minimum exposure in an additional language with studies ranging between 10% and 40%. These findings demonstrate not only a lack of consensus on the amount of language exposure in an additional language needed to be considered bilingual, but also that there is overlap in the maximum amount of additional language exposure needed to be considered monolingual and the minimum amount needed to be considered bilingual (e.g., 10–35%). Thus, an individual exposed to an additional language 10–35% of the time may be classified as either monolingual or bilingual according to published criteria, putting into question the validity of these criteria for multilingual research. While Rocha-Hidalgo and Barr (2023) included only investigations of monolingual vs. bilingual learning infants, the same issues are likely present and to a larger extent when attempting to classify learners exposed to more than two languages.

We would also like to point out that although the majority of researchers have used a minimum of 20% or 25% exposure to an additional language as the criterion for defining bilinguals, no research has established a threshold for amount of exposure necessary for multilingual development. This criterion is frequently attributed to a study by Pearson et al. (1997); however, their study did not actually provide evidence for a threshold. Quite the opposite: they provided evidence that even with less than 20% exposure to an additional language, children were still reported to produce words in that language, and the positive correlation between exposure and words produced was still significant for this group with less than 20% exposure. This finding led the researchers to explicitly conclude that “there does not appear to be a threshold effect” of exposure on vocabulary production (Pearson et al., 1997, p. 51)^1^. This provides further indication that percent exposure metrics may not be an accurate indicator to classify and identify multilingual environments.

**Figure 1:**
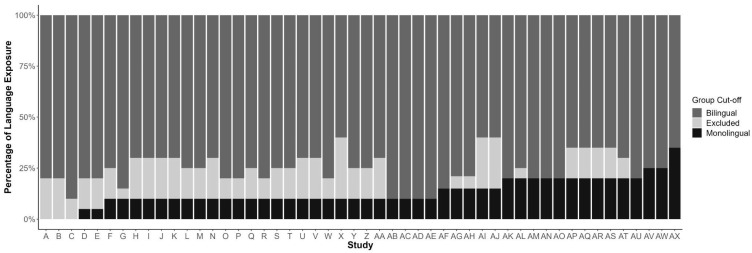
Variability and Exclusionary Practices in Monolingual vs. Bilingual Definitions. Note. Variation in the percent of language exposure cut-off criteria across studies included in Rocha-Hidalgo and Barr (2023). This figure illustrates the percentage of language exposure required for a child to be considered monolingual or bilingual, as well as the “gap” between these values. Children whose language exposure falls within this “gap” were excluded from the research.

**(2) Exclusion of some multilingual experiences.** Using percent exposure metrics to classify the monolingual and multilingual environments can lead to exclusionary practices that thwart our understanding of the diversity of multilingual experiences. Leveraging data from Rocha-Hidalgo and Barr (2023)^2^, we probed 53 studies including mixed monolingual and bilingual infant samples that used percent exposure cut-offs as determining criteria for classification into the two groups. Our analyses revealed that the majority of these studies presented a gap in inclusion criteria (*n* = 49; 92.5%). On average, these studies required a maximum of 13.16% (*SD* = 6.67; range: 0–35) exposure to an additional language to be considered monolingual and a minimum of 24.47% (*SD* = 7.68, range: 10–40) exposure to an additional language to be considered bilingual. Children who experience an additional language between these values are not considered monolingual or bilingual and excluded from participation (see Figure 1).

We then examined an additional 45 studies including bilingual samples only, revealing that the average minimum cut-off to be considered bilingual was 25.56% exposure (*SD* = 7.78, range: 10–40), similar to studies including monolingual and bilingual samples, *t*(93) = 0.691, *p* = 0.491. This finding illustrates that the gap is pervasive and remains similar across studies with mixed monolingual and bilingual samples and bilingual-only samples, showcasing that the monolingual bias has translated to research with bilingual-only participants.

Who are the children in the gap? Why are we excluding them from our research? And what do we miss by excluding them? As previously mentioned, there is some evidence that even small amounts of language exposure to an additional language contributes to some knowledge in that language (e.g., M. A. Castellana & Benitez, 2025; Hoff et al., 2012; Parra et al., 2011; Pearson et al., 1997). Excluding children who do not fit “nicely” into the monolingual or multilingual category based on arbitrary metrics and low percent exposure has essentially led us to ignore any language experiences that could potentially be contributing to building multilingual knowledge.

**(3) Capturing meaningful differences in multilingual environments.** Finally, characterizing multilingual environments via percent exposure may not capture meaningful differences across environments. Percentages themselves provide only a rough metric of input and do not capture inherent variability of individual language experiences (e.g., 25% Spanish language exposure may mean hearing 1000 daily words for one individual and 5000 for another individual; Marchman et al., 2017; Orena et al., 2019). More critically, focusing only on quantity metrics constrains how we characterize children’s actual language experiences (as recent proposals have indicated with regards to monolingual environments; see Masek et al., 2021; Rowe & Snow, 2020). By focusing on only quantifying what percent of the time children are exposed to their two languages, or how many words they hear, we miss the rich social, cultural, and linguistic structure present in children’s experiences with their multiple languages across contexts, activities, time, and social partners.

### 1.3. Moving Away from Percent Exposure and Working to Rebuild the Foundation

Taken together, much of previous research on language development has overemphasized percent exposure as a key indicator of multilingual experiences and development, an artifact of the monolingual bias (Byers-Heinlein et al., 2020; Surrain & Luk, 2019). While this framework has guided our understanding of monolingual development, it constrains how we conceptualize multilingualism by treating it as a variation of monolingualism rather than a distinct experience. As a result, the field on multilingualism has been built on a narrow, fragile foundation that identifies percent exposure as the key metric of multilingual experiences. Because multilingual experiences lead to multilingual knowledge, researchers currently have little recourse but to build on this unstable foundation, defining multilingual knowledge through this limited lens. If our initial foundation begins with monolingual assumptions, our understanding of multilingualism will inevitably reproduce those same biases. Instead, we propose to rebuild the foundation, eliminating the monolingual bias by broadening back out and addressing an important basic question: What constitutes the multilingual experience? Only after answering this question can the field establish a strong foundation and move towards meaningfully identifying the features of multilingual experiences that build multilingual knowledge. To do so, we propose using a developmental systems framework, which moves away from the need to classify individuals based on specific language experiences, and instead aims to describe what are the actual experiences children have with multiple languages. The framework does this by centering the lived, multilingual experiences of individual children interacting with their social partners existing within their cultural communities across everyday places, activities, and time.

## 2. Toward a Developmental Systems Framework of Multilingual Experience

**Figure 2:**
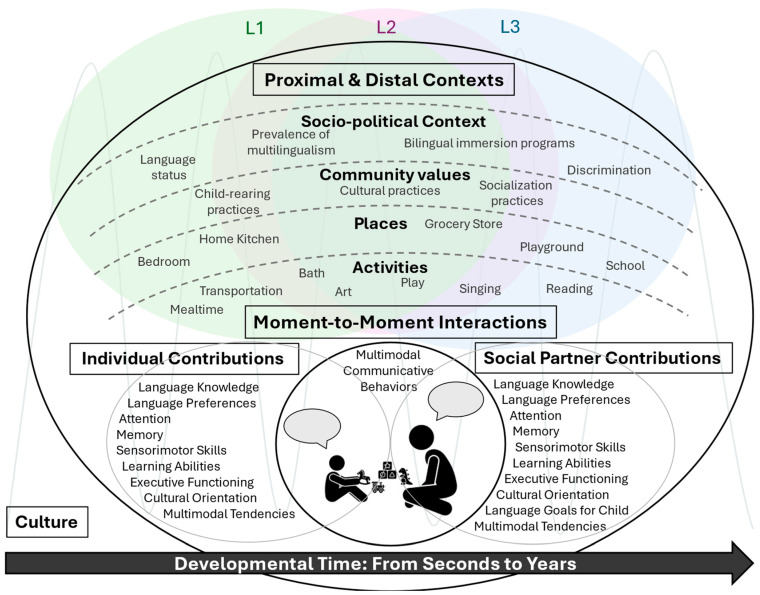
A Developmental Systems Framework of Multilingual Experience. Note: This framework centers moment-to-moment interactions (here, between the child, the social partner, and the environment) and the multimodal communicative behaviors exchanged together with languages, scaffolded by the individual’s and social partner’s contributions. All interactions are embedded within an activity (e.g., play) situated within a place (e.g., living room), structured within community values and socio-political contexts. All interactions and contexts are shaped by culture and change over developmental time. In a multilingual environment, as depicted, multiple languages can be experienced independently or intermixed across proximal and distal contexts as well as time. We acknowledge that only some contributions and contexts are listed here.

Building on previous calls to reframe cognitive and language research for racialized and minoritized communities (Figueroa, 2024; García et al., 2021; López et al., 2021; masked for review), we bridge three theoretical perspectives, the Bioecological Systems Theory (Bronfenbrenner, 1979), the Integrative Model of Minority Child Development (García-Coll et al., 1996; Vélez-Agosto et al., 2017), and the Dynamical Systems Theory (Thelen & Smith, 1996) and propose a developmental systems framework to understand multilingual experience (see Figure 2). Although each of these theories has been applied in isolation to describe multilingual experience or learning (e.g., De Bot et al., 2007; Titone & Tiv, 2023; Weisleder et al., 2024), here, we add to this prior work by bridging these theories together and considering children’s development as a central feature.

The Bioecological Systems Theory provides the foundation for our framework, highlighting that children’s multilingual experiences can be influenced by proximal factors that the child directly interacts with (e.g., family members) and distal factors that shape children’s development indirectly (e.g., policies that affect access to a high quality and equitable education; Bronfenbrenner, 1979). The Integrative Model of Minority Child Development contextualizes the distal and proximal layers by making salient the factors that shape multilingual experiences for minoritized children, such as social position assigned to class, race, ethnicity, languages, and cultural group membership (García-Coll et al., 1996). Specifically, for some learners, multilingualism can be associated with marginalization. For example, minoritized languages, compared to majoritized languages, are often less valued and marginalized, leading to inadequate resources or support for exposure to the minoritized language (Ding, 2023; Flores et al., 2021). Further, children may be exposed to multilingual input because they are members of a racial/ethnic minoritized group (e.g., if they grow up in an immigrant household). Thus, we cannot consider multilingual exposure without considering the factors that impact minoritized children’s development. Additionally, the Dynamical Systems Theory allows us to zoom in to the proximal layer and point out the ways that multilingual experience emerges from the complex interaction between sensory, motor, cognitive, and social systems that guide children’s moment-to-moment interactions with their environments and social partners (Li & Benitez, 2025; Thelen & Smith, 1996). We also consider the recent perspective that culture permeates all levels of the system, both distal and proximal (Vélez-Agosto et al., 2017). It is instantiated in everyday activities, routines, practices, and social interactions that the child engages with, as well as in the broader social and political contexts that indirectly influence development. Finally, across all layers, the framework incorporates the idea that languages can be experienced separately or together through intermixing (M. A. Castellana et al., in press-b). In the next section, we narrow in on each component of the framework to identify how each layer shapes multilingual environments and opportunities for learning.

### 2.1. Interaction Dynamics

At the center of our framework are the interactions that children have with their environment, including with social partners, who can provide rich learning opportunities for children to experience each of their languages. Interactions with social partners involve the temporal coordination of moment-to-moment behaviors (e.g., speech in multiple languages, actions, emotions) within and between individuals (Abney et al., 2017; Kosie & Lew-Williams, 2024), which can support communication and the co-construction of experiences that incorporate the use of multiple languages. Every moment is the result of the configuration of behavior that occurred in moments prior, the individual level factors that each partner brings to the table, and more distal factors that shape the contexts within which the interactions unfold (Pace et al., 2017). Language and behavior coordination between individuals across moments can drive children’s experiences with their languages. Finally, variation in the interaction between the child and their environment is a central feature—the coordination of behaviors and languages can vary within individuals (across time, context, and social partners) as well as across individuals. Different cultural groups emphasize different actions, activities, and practices, generating potentially different or similar direct experiences across languages for the child (e.g., Tamis-LeMonda et al., 2019). Understanding multilingual experience requires documenting both the content and timing of features present in interactions that shape children’s own experiences with their languages.

### 2.2. Contributions of the Individual

Each social partner can be characterized by individual-level factors that shape their contributions to the interaction. These include cognitive and sensorimotor capacities, motivation, language knowledge, prior experiences, and cultural knowledge and practices. For example, children’s own executive functioning skills, attention, memory, and language knowledge (developed from interactions) can shape subsequent interactions with a social partner (Song et al., 2014). Specific to multilingual contexts, each social partner also brings language preferences, attitudes, and practices, which can guide and shape how multiple languages are used during interactions (Altman et al., 2024; Bridges & Hoff, 2014; Dewaele et al., 2020). For example, more knowledgeable social partners (such as parents, older siblings, grandparents, and teachers) may have established language values and preferences for their own language use and for their child’s language development that guide how they share each language with their child during moment-to-moment interactions (e.g., family language policies, such as one-parent-one-language) (De Houwer & Nakamura, 2021; Lee et al., 2015; Schwartz et al., 2011). Further, parents who are strongly oriented to the heritage culture may hold a desire for heritage language maintenance (Ochoa et al., 2023), increasing their use of the heritage language with their child (Tsai et al., 2012). Parents can also infuse cultural values into their conversations with their child, which may influence children’s uptake of their different languages (M. A. Castellana & Benitez, 2025; Tamis-LeMonda et al., 2020). Importantly, children are actively developing their own language preferences, values, and attitudes as they learn from their social partners (Halpin et al., 2024). Thus, the child actively contributes to interactions which in turn shape the input they receive from their partners.

### 2.3. Proximal Contexts

The next layer of our framework encompasses the proximal contexts within which interactions occur (Casillas, 2023; Rowe & Weisleder, 2020). These include the different contexts within which children directly experience their multiple languages, such as across different activities (e.g., reading, play, mealtime, bathtime) or places (e.g., the child’s neighborhood, school, doctor’s office, grocery stores). Engagement in various activities or at different places influences the language exchanged within these contexts (i.e., experiencing more food-related talk when baking or in the kitchen) (Custode & Tamis-LeMonda, 2020; Tamis-LeMonda et al., 2019). For some children, exposure to their languages may be constrained by activity (e.g., meal times may always be in one language, while academics may always be in a different language), whereas other children may experience their languages intermixed for different activities.

### 2.4. Distal Contexts

The outer layer of our framework depicts the distal factors that can shape both individual-level factors and interactions between children and their environment (Rowe & Weisleder, 2020). More distal factors that can influence children and their interactions with the environment indirectly are also important, such as the availability of dual language programs in schools, socio-political climate, and marginalization of or the values and power assigned to certain languages. For example, funding for libraries to have multilingual books or offer multilingual programming can impact the availability of these programs for multilingual families. Importantly, these features can have direct and indirect consequences on the individual and/or the interaction. Lack of support for multilingual activities, experiences, and education in a community may make it more difficult for a parent who wishes their child to be multilingual to expose their child to multiple languages.

### 2.5. Developmental Time

Lastly, time influences all layers within our framework. At the level of the interaction, time is a critical feature—it organizes the coordination of behaviors, and any one moment in which the child is interacting with their environment cannot be understood without assessing prior moments. At the level of the individual, as children age, their language, cognitive, socio-emotional, and sensorimotor abilities also develop. These skills in turn influence children’s ability to interact with their environment and their social partners, and in turn, what input is elicited. On the part of the social partner, their language use may also be developing over time (e.g., immigrant parents’ learning the societal language; bilingual parents becoming more confident in their least dominant language), which can shape their language use with the child. Goals and attitudes towards multilingualism can also change, for both the child and the social partner, driving how each incorporate their languages during interactions. With regard to the distal factors, time can bring about change in policy, institutional practices, and support or resources for multilingual use. These changes can then have cascading and indirect consequences on the experiences that a child has with their multiple languages.

## 3. Future Research Directions with This Framework

Our model outlines new areas of research to understand the multilingual experience that have not yet been explored. Here, we propose several possible future directions that the framework highlights are necessary to capture the moment-to-moment, multimodal, interactional, contextual, and longitudinal nature of multilingual experience.

### 3.1. Interaction Dynamics: Moment-to-Moment Temporal Coordination of Multimodal Behaviors

At the center of our framework are interaction dynamics, which make salient the coordination of multiple languages and behaviors moment-to-moment across time (Abney et al., 2025). However, research on multilingualism typically only focuses on the languages produced during interactions, with less attention on the additional multimodal behaviors that frequently accompany language, such as action, gesture, and emotion, as emphasized recently in research with monolingual infants (e.g., Kosie & Lew-Williams, 2024). We have yet to determine how multimodal behaviors are coordinated with multiple languages. Are multimodal behaviors used similarly across languages? Recent research has begun to address these questions, providing evidence that negative emotional affect predicts higher subsequent codeswitching (in a sample of U.S. Chinese-English bilingual parents and their 7-year-old child; Williams et al., 2020) and that children may gesture differently based on the language context (in a sample of 18-month-old U.S. Spanish-English learning toddlers; Gámez et al., 2025). These findings indicate that multimodal behaviors may be tightly coupled with multilingual language use in systematic ways, warranting further exploration into how language and behaviors co-occur.

Further, specific analytic methods, such as dynamic structural equation modeling, (Asparouhov et al., 2018; McNeish et al., 2024) and cross-recurrence quantification analysis (Abney et al., 2017; Dale & Spivey, 2006; Duong et al., 2024), can help quantify interaction dynamics, determining both the degree and pattern of coordination across behaviors. However, only one study to our knowledge has assessed interaction dynamics in multilingual contexts, suggesting that culture may be a stronger predictor of the dynamics of multimodal communication than bilingualism (Gampe & Hartmann, 2020), given that different cultural groups emphasize different behaviors during interactions with their children (Cote & Bornstein, 2021; Gratier, 2003). These findings point to the importance of considering culture and language background together when assessing interaction dynamics.

### 3.2. Contributions of the Individual: Identifying Variation in Input Across Social Partners

Our framework highlights that different individuals bring unique characteristics to an interaction that can shape how they contribute language(s). Interactions with an age- or knowledge-similar peer (e.g., sibling, friend) facilitate different opportunities for language use and learning than interactions with a more knowledgeable other (e.g., grandparent, teacher; Bulgarelli, 2026), especially as each may have different goals and motivations for their use of multiple languages with the child. For example, research has documented variations among children’s, mothers’, and fathers’ use of infant-directed speech (Ferjan Ramírez et al., 2022; Pancsofar & Vernon-Feagans, 2006; Weppelman et al., 2003), perhaps reflecting variations in knowledge of how infant-directed speech supports language development. In multilingual contexts, different social partners may use their languages in different ways, shaping children’s exposure and learning opportunities (e.g., Ferjan Ramírez et al., 2022). For example, Spanish–English dual language learning toddlers in the U.S. have been found to hear more English from their school-aged siblings than other household members (Bridges & Hoff, 2014). Further, multilingual parents may hold different language and cultural values (e.g., enculturation) and goals (e.g., heritage culture or language maintenance) for their child that shape their language use (e.g., heritage language use only, one-parent-one-language approach) to support their goals for their child’s multilingualism (King & Curdt-Christiansen, 2021; Mercado Ramos et al., 2025; Tsai et al., 2012). Yet, the majority of prior work on multilingual interactional contexts has focused on maternal input, limiting our ability to understand how various social partners’ characteristics, experiences, motivations, and goals shape their use of multiple languages with children. Addressing this gap is essential for advancing a comprehensive understanding of how social partners contribute to multilingual learning opportunities in interactional contexts.

### 3.3. Proximal Contexts: Measuring Variation in Language Learning Opportunities Across Activities and Places

Capturing how multiple languages are experienced across contexts is crucial for understanding how multilingual knowledge emerges from diverse experiences. Here, we refer to contexts at a proximal level as the activities and places through which young children may experience their languages directly. Recordings of monolingual parent–child dyads engaging in natural everyday activities at home have shown that routine-oriented and activity-related talk is closely linked with the specific activity and room in which the interaction occurs (Custode & Tamis-LeMonda, 2020; Tamis-LeMonda et al., 2019). Daylong recordings in the home have also provided a foundation for understanding how children can experience language across an array of activities that occur over the course of the day (Bergelson et al., 2019; Casillas, 2023; Orena et al., 2019). Yet, it is currently unclear whether and how multilingual language use varies across activities, such as reading, play, mealtime, or bathtime. Are certain activities likely to elicit one language more than other languages or balanced input? Is language mixing more likely to occur for some activities than others? Additionally, contexts beyond the home or school may invite different language use, a research area currently understudied (Larson et al., 2020; Welsh & Hoff, 2021). For example, visiting a grocery store in a Spanish-speaking neighborhood in the U.S. may expose children to food words in Spanish (e.g., albahaca) without experiencing the English equivalent, resulting in distributed knowledge. Identifying how children experience their different languages beyond the typical contexts studied (home and school) could shed light on how multilingual knowledge may be impacted by other frequently visited places (e.g., grocery store, pediatrician, neighborhood playground).

### 3.4. Distal Contexts: Measuring Variation in Language Learning Opportunities Across Community Values and Socio-Political Contexts

At a broader level, communities vary in how they value and use different languages, often in response to social, cultural, and political pressures. Cultural values and beliefs can shape socialization and child-rearing practices that impact how individuals interact with children (e.g., the amount of child-directed speech used; Casillas et al., 2020). Language ideologies about whether multilingualism is viewed as normative or negative can shape the extent to which multiple languages are used with a child, which in turn, influence how children develop their own language identities, attitudes toward, and use of multiple languages (e.g., Fernández, 2006; Halpin et al., 2024). Speakers of a minoritized language may also face language discrimination for speaking their native language, which has been documented in both children and their parents (Ayón, 2016; Eyal et al., 2023). However, the extent to which they experience this discrimination, and how linguistic discrimination may shape exposure to multiple languages, is not well understood.

Further, the sociopolitical context regarding multilingual development (e.g., in the U.S, the availability of bilingual immersion vs. English-only educational instruction, early English Learner identification, and services; M. Castellana, 2024) likely produces cascading impacts on children’s multilingual experiences and development (e.g., Combs et al., 2005), a research area that is severely understudied. For example, does the availability of programs and activities that support learning different languages (e.g., bilingual immersion programs; after-school classes for different languages) contribute to more multilingual community norms, increasing children’s exposure to multiple languages in the community? Addressing these questions is critical for understanding the direct and indirect impacts of socio-political environments on children’s multilingual exposure.

### 3.5. Developmental Time: Understanding Change at Different Timescales Across Layers

Time shapes individuals and interactions at the micro-level, as well as contexts and environments at the macro-level. Within the timescale of interactions, research should identify how interactional behaviors and the use of multiple languages unfold together temporally, as described above. Research should also aim to identify how multilingual exposure ebbs and flows over the course of minutes, hours, days, and weeks. Prior research in monolingual samples, using recordings from 5 min to 2 h (Tamis-LeMonda et al., 2017, 2019) or over the course of the day (Bang et al., 2023; Casillas et al., 2020; Orena et al., 2019) have documented how exposure shifts from high-density moments, in which a lot of child-directed or other-directed speech occurs, to moments of silences. Ecological momentary assessments, a method for data collection of participants’ behaviors in real time (Shiffman et al., 2008; e.g., Place & Hoff, 2011), can be implemented to gather information about the activities, places, and speakers in an environment, revealing patterns of experience in a day or over multiple days. When used in combination, these methods can prove to be powerful tools for revealing children’s real-world experience with language (Nencheva et al., 2026). For children who experience multiple languages, what are the temporal dynamics of multilingual exposure and language mixing? Is the temporal pattern of exposure in one language similar or distinct from the temporal pattern of the other language(s)? It will be important for future research to conduct dense sampling of language exposure over different time scales (i.e., minutes, hours, days, or weeks) to gather information about the temporal dynamics of multilingual exposure (e.g., most frequently said words during a current activity; the number of speakers in an environment), showing how multiple languages may be experienced in real-time.

At longer timescales, identifying how exposure to multiple languages decreases, increases, or remains the same over the course of months or multiple years can help identify longer-term exposure trajectories. Only a few studies have explored how exposure changes over longer time periods, focusing on Spanish-English bilingual children in the U.S., finding that children gradually hear more English across childhood (M. A. Castellana et al., in press-a; Luo et al., 2020; Welsh & Hoff, 2021). Longitudinal variations in language exposure over time ultimately shapes children’s developmental trajectories in each language (M. A. Castellana et al., in press-a; Luo et al., 2020). Pinpointing the natural short and long-term temporal dynamics of multilingual exposure can help identify the optimal structure that promotes strong multilingual learning.

### 3.6. Culture: Comparing Multilingual Experiences Across Communities

Understanding how multiple languages are experienced across various moment-to-moment interactions, social partners, activities, places, socio-political environments, and time requires assessing different multilingual groups from different regions and cultures to provide representative and culturally grounded findings. Without sampling across diverse cultural and sociolinguistic environments, research risks drawing conclusions that reflect only a narrow set of experiences and may overlook how multilingualism is experienced in different places across the globe. These comparisons can occur within country. For example, does a Spanish-English bilingual learning child growing up in the Southwest United States experience their two languages similarly to a Spanish-English bilingual learning child in the Midwest United States, or a Mandarin–English bilingual learning child on the West Coast United States? These comparisons can also occur across countries. For example, how do children experience multiple languages if growing up in India, Malawi, Mexico, or Singapore? Understanding consistency and variability of multilingual exposure across the globe can shed light on the factors that matter most for multilingual development. This research could then support parents raising multilingual children and scaffold culturally sensitive policy development regarding multilingualism (e.g., increasing the number of and funding for bilingual teachers and programs) for different children around the world.

## 4. Conclusions

We have argued that a pervasive monolingual bias has infiltrated current research on multilingualism, producing a limited understanding of the multilingual experience by reducing multilingualism to the percent of exposure to each language. Rebuilding the foundation through which research examines multilingualism requires stepping back and addressing one basic question: what is the multilingual experience?

We proposed a developmental systems framework that seeks to move the field toward a better understanding of the multilingual experience by centering the lived experiences of multilingual children and their interactions with social partners across modalities, activities, places, and time, embedded within their cultural practices, values, beliefs, and communities. Reorienting research exploring the multilingual experience within this framework will promote work that is more ecologically valid and theoretically grounded, which will subsequently yield a more accurate understanding of how early multilingual knowledge emerges.

We also offer a few additional recommendations when working with young children growing up in a multilingual environment. First, regardless of whether multilingualism is the focus of a research study, we recommend using multiple metrics beyond percent exposure to describe learners’ experience with multiple languages, such as with whom and where they experience their different languages (e.g., Byers-Heinlein et al., 2020; Place & Hoff, 2011). Second, we recommend that research not use percent exposure metrics to classify individuals as monolingual or multilingual, given that this metric is arbitrary and does not meaningfully capture language experiences. Since children’s language experiences are rapidly evolving in early childhood (e.g., Luo et al., 2020), it may be premature to classify a child as monolingual or multilingual. Instead, we urge researchers to describe learners based on the opportunities they have to be exposed to multiple languages, adopting terms such as “bilingual or multilingual learner,” “bilingually- or multilingually-exposed children,” or “children receiving bilingual or multilingual exposure.” Finally, we urge researchers to stop excluding infants and children from their studies based on whether children receive a specific amount of multilingual exposure. Doing this to “clean” the research design or delineate categories perpetuates the exclusion of important linguistic variation that is naturally present in children’s real-world environments. In fact, the children whom research has excluded (because they are in the “gap” or have multilingual exposure) may provide novel insights into multilingual emergence to move the field forward.

Finally, our framework can guide the use and refinement of questionnaires developed to assess children’s language backgrounds, including those used as part of diagnostic evaluations of language impairments (e.g., Alberta Language Development Questionnaire, Parents of Bilingual Children Questionnaire; Paradis et al., 2010; Tuller, 2015). These tools do rely on quantitative metrics (e.g., estimates of language use and exposure, timing of milestones), which provide valuable information. We believe that this information forms just one part of the larger picture of potential experiences a child could have with their languages. Our framework makes salient the complex ways in which multiple languages could be experienced, and thus, assessments should not stop at quantitative metrics. Our model can guide future refinements to these tools by emphasizing the measurement of multilingual experience across partners, activities, and places, as well as measuring patterns and variability across time and interactions. These changes would preserve the validity of these instruments while aligning them more closely with the lived experiences of multilingual children via our proposed framework (e.g., Castro et al., 2020).

We call for the field to center the multilingual experience by reframing our theoretical perspectives from a monolingual to a multilingual lens. Through the use of our proposed developmental systems framework of multilingual experience, the field can strive to decolonize language and cognitive development research and better understand the diverse experiences that constitute language environments across the world.

## Data Availability

The data presented in the current study are from Rocha-Hidalgo and Barr (2023) and are openly available in Open Science Framework at https://osf.io/5fhrb (accessed on 2 May 2024).

## Notes

1 The criterion of 20–25% likely came from a point later in their Discussion section, in which Pearson et al. (1997) described a practice by their lab staff to “recommend that children whose exposure was less balanced than 75:25 not be considered for future bilingualism studies.” This was due to reports that children with less than 20–25% exposure to an additional language had difficulty producing enough language samples upon request or spontaneously during language assessments at later timepoints in the study. The authors then propose that there may not be a threshold for the effects of exposure on vocabulary knowledge but there may be one for producing utterances (syntactic knowledge) and admit that this would be a fruitful area of future investigations (bottom of pp. 55–56).

2 Assessment and inclusion of the data in this manuscript was done with the authors’ permission.
